# Supplementary material for: Bibacillin 1: a two-component lantibiotic from Bacillus thuringiensis
Source: RSC Chem Biol. 2024 Sep 4;5(10):1060–73. doi: 10.1039/d4cb00192c (PMC11385697; doi:10.1039/d4cb00192c)
Supplement: CB-005-D4CB00192C-s001 [file CB-005-D4CB00192C-s001.pdf]

Supplementary Information (SI) for RSC Chemical Biology.
This journal is © The Royal Society of Chemistry 2024

Supporting Information

Bibacillin 1: A two-component lantibiotic from *Bacillus thuringiensis.*

Ryan Moreira,[1] Yi Yang,[1] Youran Luo,[1] Michael S. Gilmore,[2] and Wilfred van der Donk[1,*]

[1]*Department of Chemistry and Howard Hughes Medical Institute, University of Illinois at Urbana-Champaign, Urbana, IL, 61822, USA*

[2] *Departments of Ophthalmology and Microbiology, Harvard Medical School, Boston, MA 02144, USA*

Table S1. Strain information and growth conditions	S2
Table S2. List of primers	S3
Table S3. Sequences of codon optimized gene fragments	S3-4
Table S4. Accession numbers	S5
Fig. S1. LCMS-MS analysis of Bib1β_P	S6
Fig. S2. Solution phase hemolytic activity of bibacillin 1	S7
Fig. S3. Additional continuous variation experiments	S8
Fig. S4. Structure of cacaoidin	S8
Fig. S5. Bib1β_P and Bib1β S16A CylR1/R2 triggering	S9
Fig. S6. SDS-PAGE analysis of bibacillin 1	S9
Fig. S7. R-HPLC traces of purified Bib1α, Bib1β, Bib1β_P, Bib1β_{Dha}, and Bib1β S16A	S10
Fig. S8. HRMS spectra of purified Bib1α, Bib1β, Bib1β_P, Bib1β_{Dha}, and Bib1β S16A	S11
Table S5. Comparing high resolution mass to expected masses for Bib1α, Bib1β, Bib1β_P, Bib1β_{Dha}, and Bib1β S16A	S12
References	S12

Table S1. Strain information and growth conditions

Strain	Source	Media	Temperature (℃)
Lactococcus lactis subsp. cremoris NZ9000	Kuipers et al.[1]	M17+0.5% glucose	30
Micrococcus luteus	ATCC[a] 4698	Tryptic Soy Broth (TSB)	30
Bacillus subtilis 168	Helmann et al.[2]	Lysogeny broth (Miller)	37
Bacillus thuringiensis subsp. pulsiensis	BGSC[b] 4CC1	3 nutrient broth (3N)	30
Escherichia coli BL21 (DE3)	UIUC-CMF[c]	Lysogeny broth or Terrific broth	37
Enterococcus faecium	ATCC 19434	BHI	37
Enterococcus faecalis	ATCC 19433	BHI	37
Enterococcus faecalis	ATCC 29212	BHI	37
Pseudomonas aeruginosa PA0162	Manoil et al.[3]	3N	37
Staphylococcus aureus NRS3	NARSA[d]	TSB	37

[a]ATCC: American type culture collection. [b]BGSC: Bacillus genetic stock center. [c]UIUC-CMF: University of Illinois at Urbana-Champaign cell and media facility. [d]NARSA: Network on antimicrobial resistance in *Staphylococcus aureus*.

Table S2. Primers used for molecular biology.

**Name**	**Sequence**
Bib1A1 FP	gtttaactttaataaggagatataccatgccacatcaccatca
Bib1A1 RP	tttgattcatggtatatctccttgaatcttagcagtgcatccggct
Bib1A1Mfrag1 FP	actgctaagattcaaggagatataccatgaatcaaaatacaagcataaaac
Bib1A1Mfrag1 RP	ttgccgttcagctttggcacgttgttatcgtttatatcagccataactt
Bib1A1Mfrag2 FP	atggctgatataaacgataacaacgtgccaaagctgaacggcaa
Bib1A1Mfrag2 RP	cggtttctttaccagactcgagctaatatttgataggcttttctaaggtcaa
Bib1A1 pRSF FP	cttagaaaagcctatcaaatattagctcgagtctggtaaagaaaccg
Bib1A1 pRSF RP	tgatggtgatgtggcatggtatatctccttattaaagttaaac
Bib1A2 FP	gtttaactttaataaggagatataccatgccgcatcatcac
Bib1A2 RP	ttatgcttgtattttgattcatggtatatctccttgaatctcaacaataaattttggagat
Bib1A2Mfrag1 FP	tttattgttgagattcaaggagatataccatgaatcaaaatacaagcataaaacccc
Bib1A2Mfrag1 RP	ttgccgttcagctttggcacgttgttatcgtttatatcagccataactttttttgcg
Bib1A2Mfrag2 FP	atggctgatataaacgataacaacgtgccaaagctgaacggcaa
Bib1A2Mfrag2 RP	ttctttaccagactcgagctaatatttgataggcttttctaaggtcaa
Bib1A2 pRSF FP	ttgaccttagaaaagcctatcaaatattagctcgagtctggtaaagaa
Bib1A2 pRSF RP	gtgatgatgcggcatggtatatctccttattaaagttaaac
Bib1 A2 SSM S64C RP	ccgccaatggcacaaacaggagt
Bib1 A2 SSM S64C FP	tggcctcgcattgggtttttgtatctccaaaatttattgttga
Bib1 A2 SSM S64A RP	ccgccaatggcacaaacaggagt
Bib1 A2 SSM S64A FP	tggcctcgcattgggttttgcaatctccaaaatttattgttga

Table S3. Codon optimized gene fragments

Name	Sequence
Bib1A1	atgccacatcaccatcaccaccacatgaaccggaatcaaattattgaagaactcgctgtgaatcaccctgcgggtgcgaagctggttgaggtcagcaaggaagagcttacccgggtgtacgggggtggcgatgttcaagcagaaacttctcctgcctgtgcttccgcaatgctttatctgcaggggggttacggtgtctctcggtctgagccggatgcactgctaa
Bib1A2	atgccgcatcatcaccatcaccatatgaatcggaacgaagttattgaagagttagcagtcaatcacccagctggtgctaagttagtggaagtatcaaaagaagaattgacgcgcatttacgcgggggcgatgttcaggcagaaaccactcctgtttgtgccattggcggtggcctcgcattgggtttttcaatctccaaaatttattgttga
Bib1Mfrag1	atgaatcaaaatacaagcataaaaccccttatctctaccactctgtttaaatctctgagtatcaaagaacggattcagcttgcagataaaattgatacgattgacctcaatgaggaggaatttcagagttatcgcaataaatggaaagaagaaacgctgcttaacgatgattccatgaataagagaatacaggtcgagcaactggatgagaaactctttataaaagcgttgaaggcaagtgaaaacgaggaatggcttcaacaaaacaagctggaaccgaaccaaaatccactttggatgaattggattgaggaagcactcacccttcatcgtaataaggagcttaatgaaccagatcaactccatatttcattagcattccgaccgttcatgttatgggctaagaatcgatttaacaaattttatcaggaacacccgtcgctttcaaaaaaagttgattgggatcagttgcagtcttctattctgtataatctgattgaggccctgacaaatattggagcgcg

	cacaatcactttagaattatatattgcaaaacagttaaaagaattaacaggcaacacgccggaagaacgcttcgagtcttttgtgcggactaaacttctggactttgatgcgctggaatttatttataaagaatatccggttctgtcgcgtattttgatgatacgtactaactattatgtaaacgcaattacagaagccctgacccgttttgaggaagattgggaccagattaaccaaagtttaaaactggatgctttatcgttaaaaagtatcgccgtcggattaggggatagtcatcagcagggtcgaagcgtaatgcgctttaaatttgaaaaaaacaaagagatattatataagccgaaaccattaaccgttgctatttatttccacgagctgctggattggattaacaaaaagggttttactccaaaattgaaaggccataacattttaaacagacaaggctatgtctgggaagaatgcattcaacatcgagaatgcaaaacccaggaccagattgaaaattattacaagcggctcggtgggtatttagctatattaaacgcagtgaatggtactgatttccaccatgaaaatatagtcgcggacggcgaatttccgacgctgatcgatttagaaacgatttttcaccatcctgccaagttgaatataaagactgcagaaatcgaggctaaatacaagatcattaattcagtgttagggaccgctttgcttcctcacttatattttaaaaatgccgagggctatggtattgatatcagcggcgtgagtgtccagaaccaggaggagctgccgattccgttactccgtccggagaacgaggggactgatgagatgcgtttcgtccgcaaaaaagttatggctgatataaacgataacaac
Bib1Mfrag2	gtgccaaagctgaacggcaaagtaattggagcatctagtcacgtggattccatcatcgagggctaccagcacgcggcccagataatttttaataataaatccgaactgctgcatgaagacgggcctatagcaaaattcaaggataccgaaattagaattgttgtgcgtcccacacagtactatggcaattttctgcttgaaaaatatcatcctgattacatgcgtgactgcgtggaattagaaaaactgttggatcgactttggtttaccgtacttgacacgcgacagattccgttcgagaaacaggatttgtttaatggtgatattcccatttttacaactaagcctggatccagagatctgtttgcctcatccggtgagaaaatagaaaattattttgatcagccgtcctacgaaatcgtggttgaacgaattaaaaacttaaccctcgattctattgaagaacagagtaaatggatcgaggctagcctgagctgcaacattaaagagaaagttattgtgaaggaagctacatatctcaaagaagaattaaaagaaatcaagaccgatattttcatcgaagaggccaagaacattggttatcgtctgaaggagcaggttattcatggctcgtataatgatgccacctgggtgggtctcggtatgaattaccacaatcaatggcaagtaactgcactggattcaggattatataacggtttgtcaggcattgcaatgttcctgggataccttggtaaagtgtctggtgaagaagactttaaccggttagcaatgcagaccatggaatccatattgcagcaaccgattcaagaaaaaagctttgcatctgcgttttatgggcaggccagtcacctttatatcttatctcattttgacgccctgtatggcgaaaaccaaaaaatggaagctgtacattcaaaactctctgaacaacattgaaaagagcgtgaaaaatgaccagttctatgatttattgggcggaagtgccgggattattcaggttctgttaaatatacatgaacagtttaataatgagcaggcgttaaatatagcacaaaaatacggcaatcatttaattgaaaataaaatcgtgacagagagaggtatcgggtggtcagatccttcgagccaaatcatgctcggcggactgagccatggcacctcgggcatcgcttggtctctgcttcggcttcataagcagaccagcaaacagaagtactttgaaaccgccctggaggcgatccgatatgaccgtagcctttataacagcaagaactgtaattgggaagatttaagatgttcccaacataaatcgtctgaattcagtgcagcgtggtgtcatggtgccactggcatagggctcagtcgactgttatacctcccctacattaaagatacacttttgcaacaggagattgaaaccgcggtcagtactacagtggatgttgggatggggcgcagtcacagtctctgtcatggtgacctcggtaatagcgaactgtttcatgtggcagggaacgttctcggaagaccagagtggaatagaatggcgcacgcggtaggtatgaacaccataaaagagaaacaagaaattggaaagtacaaaacaggcgtaggtcgtcatatcgagatccctggactgtttatgggcctgtcaggaatcggataccagctcttacgtctggctaagccaaaccaagttcccagtgtcttgaccttagaaaagcctatcaaatattag

Table S4. The accession identification numbers associated with the corresponding proteins named in this work

Accession	Name
WP_098353184.1	Bib1R1
WP_000933887.1	Bib1R2
WP_098353186.1	Bib1M
WP_016078320.1	Bib1 A1
WP_016078319.1	Bib1 A2
WP_098353188.1	Bib1A
WP_098353190	Bib1B
WP_016078316.1	Bib1R

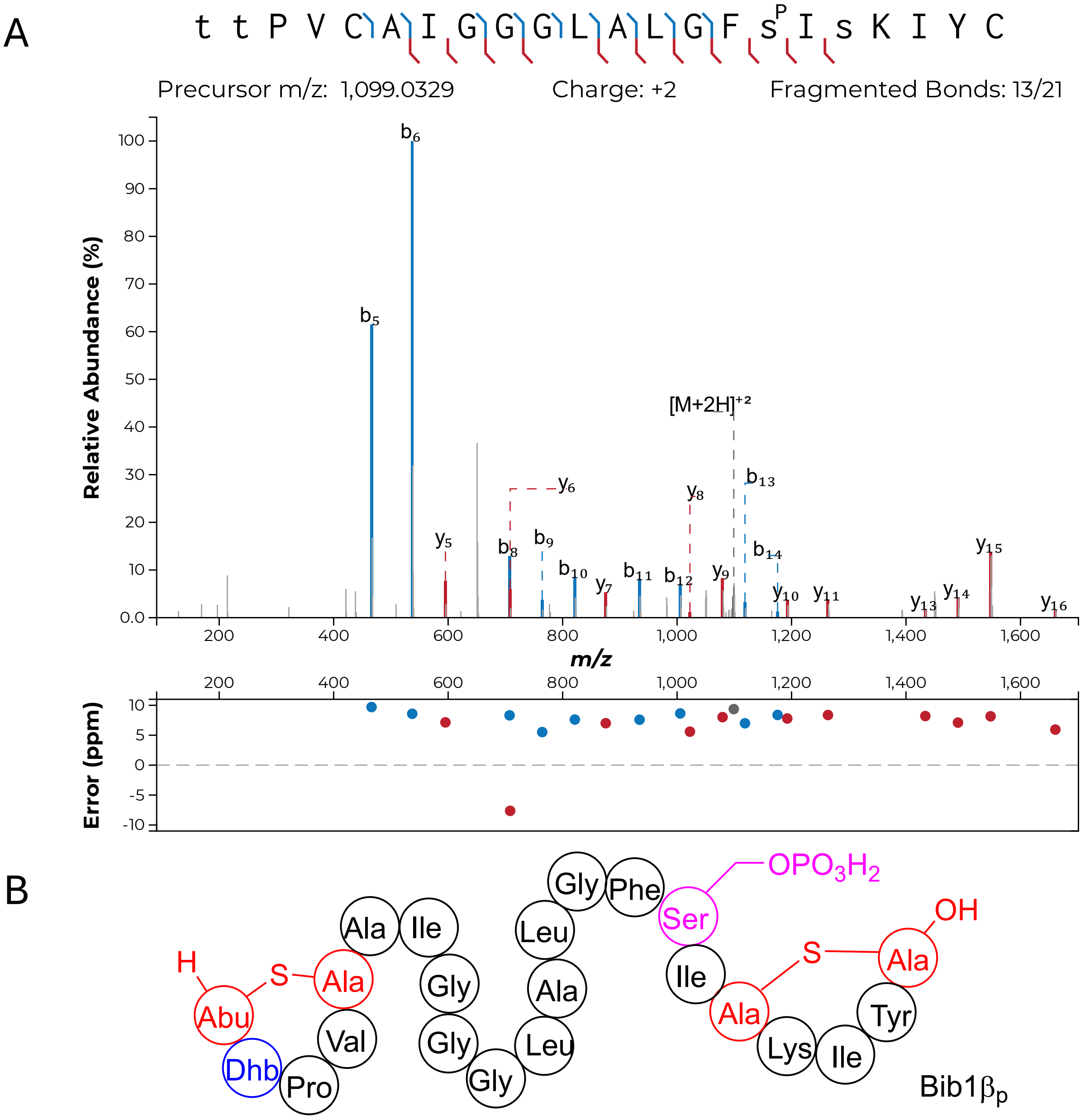

Fig. S1. LCMS-MS analysis and ring pattern of Bib1βP. A) Analysis of the fragmentation pattern produced during tandem MS-MS. Lower-case labels indicate that a residue was post translationally modified. ‘s^{P}’ indicates that a Ser was phosphorylated. All other lower-case residues were dehydrated. B) Ring pattern and phosphorylation site consistent with the LCMS-MS analysis.

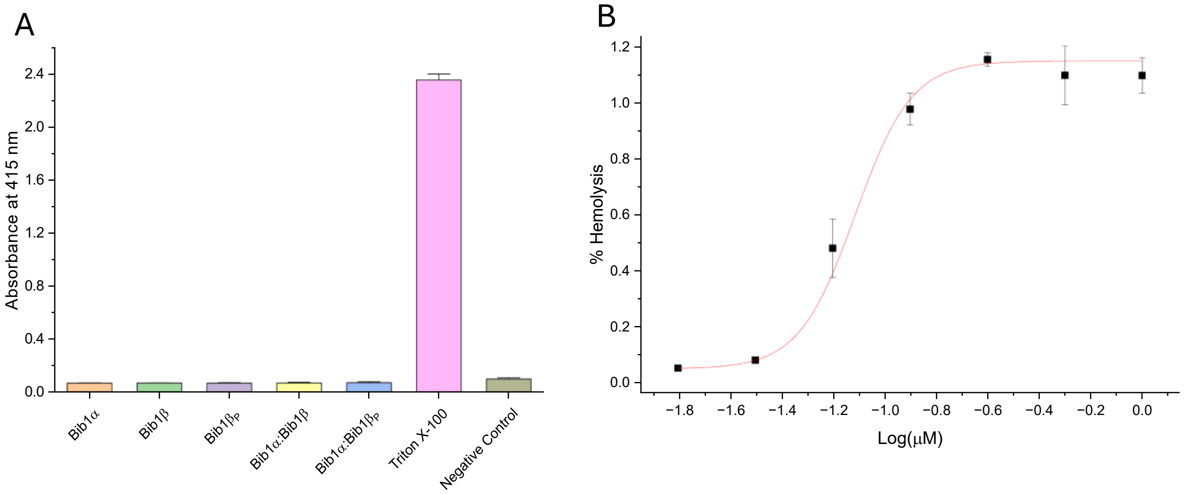

Fig. S2. Bibacillin 1 does not show hemolytic activity in solution at ≤2 μM. A) Hemoglobin release (measured by absorbance at 415 nm) after treating rabbit erythrocytes with Bib1α, Bib1β or Bib1β$_P$ individually or with a 1:1 of Bib1α:Bib1β or Bib1α:Bib1β$_P$ for 1 h at 37 ℃. As a positive control, erythrocytes were treated with 0.03% v/v triton X-100. Negative control contained only buffer. A total peptide concentration of 2 μM was used in each case. B) Extent of hemolysis after treating freshly washed rabbit erythrocytes with 1:1 of CylL$_S$":CylL$_L$" at 37 ℃ for 1 h. Data was fit to a dose response curve using Origin 7 yielding an EC_{50} = 76 ± 5 nM.

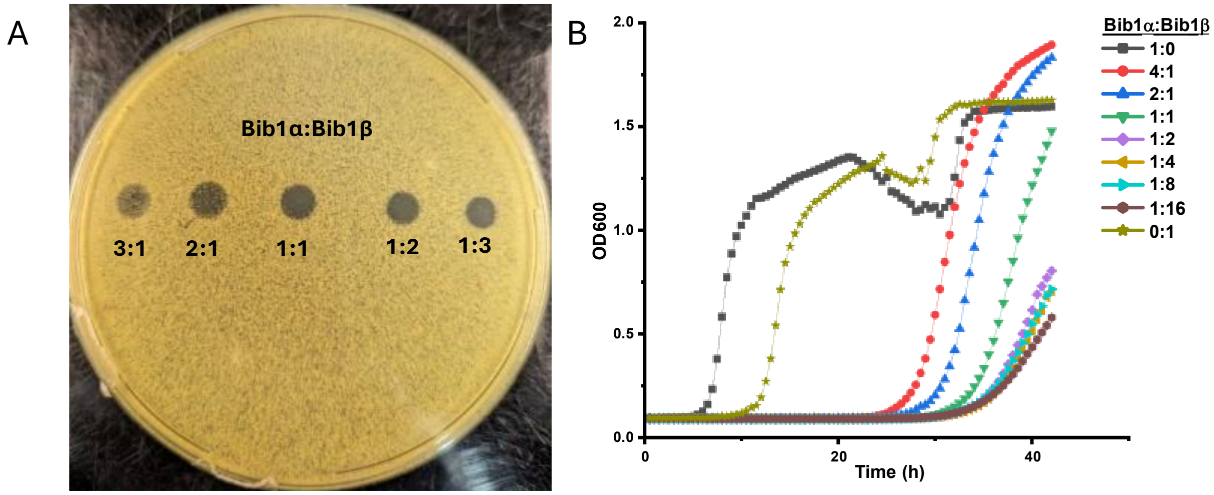

Fig. S3. Continuous variation experiments demonstrate that bibacillin 1 does not produce maximal activity at a well-defined ratio of Bib1α and Bib1β. A) Agar diffusion assay using *Micrococcus luteus* ATCC 4698 seeded soft agar. Bib1α and Bib1β were diluted to 100 µM in PBS then mixed together to produce the indicated ratios and 3 µL of each mixture was spotted on the agar plate. The total amount of peptide deposited was 300 pmol in each case. B) Growth curves collected simultaneously with those featured in Figure 4E. In this case, the total peptide concentration was kept at 5 µM.

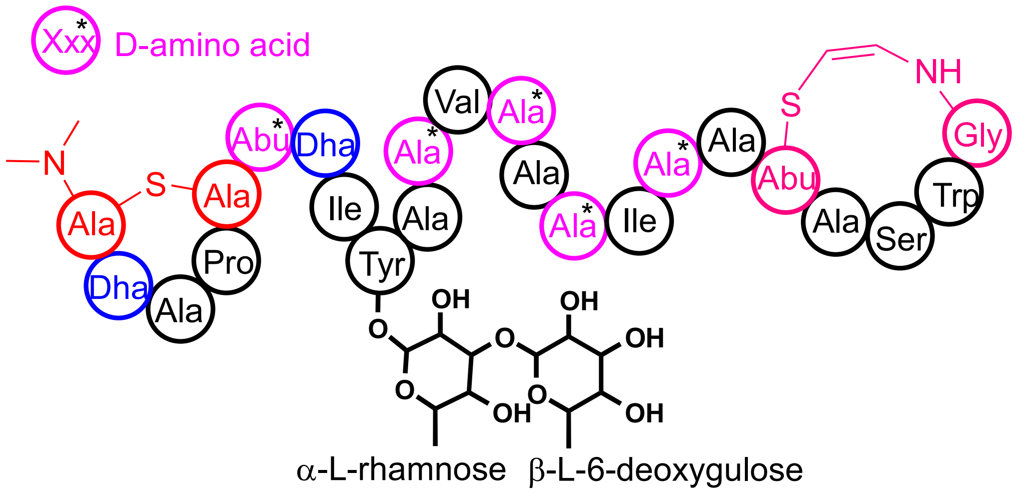

Fig. S4. The structure of cacaoidin. Post translationally modified residues are color coded. Red residues comprise an *N*,*N*-dimethylated lanthionine. Blue residues are dehydrated amino acids. Magenta residues containing a black asterisk are D-amino acids. Residues involved in S-[(Z)]-2-aminovinyl- (3S)-3-methyl]-D-cysteine are colored rose pink.

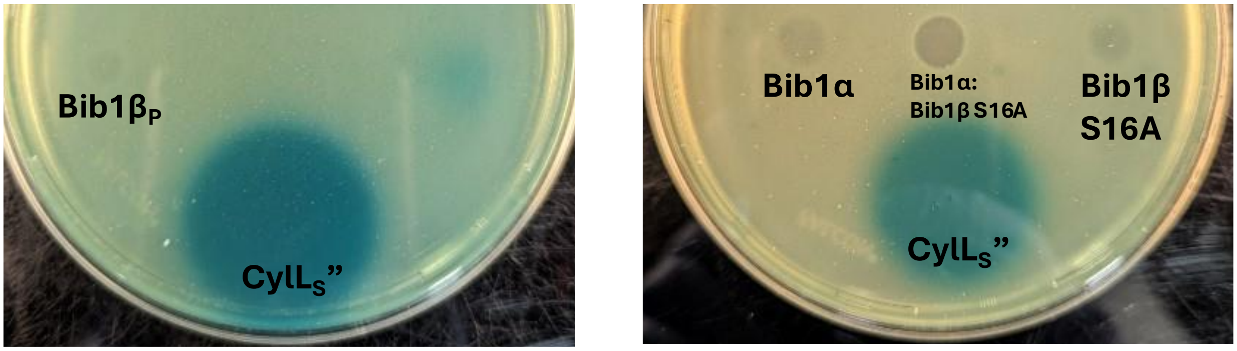

Fig. S5. Bib1βP and Bib1β S16A do not trigger the CylR1/R2 system. Plates were prepared as described in Figure 6A.

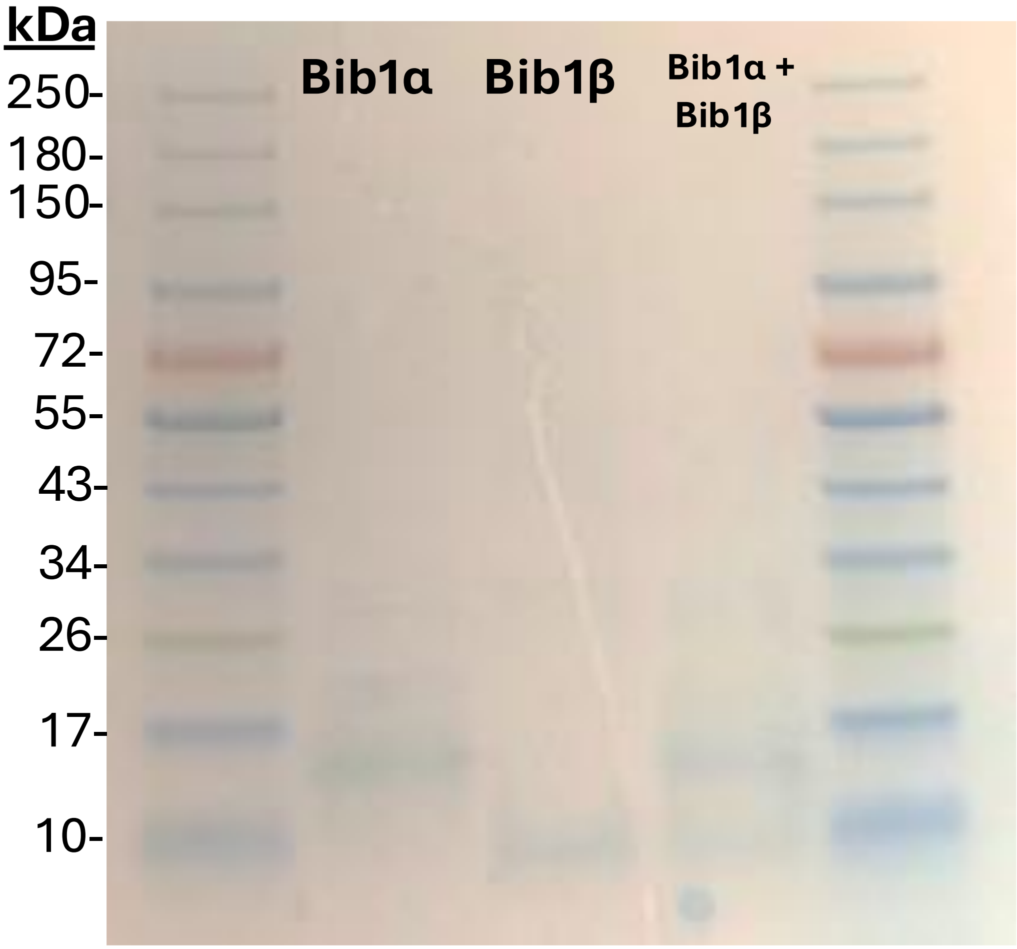

Fig. S6. SDS-PAGE of Bib1α, Bib1β or Bib1α:Bib1β (1:1). In total, 2 nmol of peptide was present in each lane. A gradient of 4-20% acrylamide concentration was used. Coomassie staining was used to image peptide species.

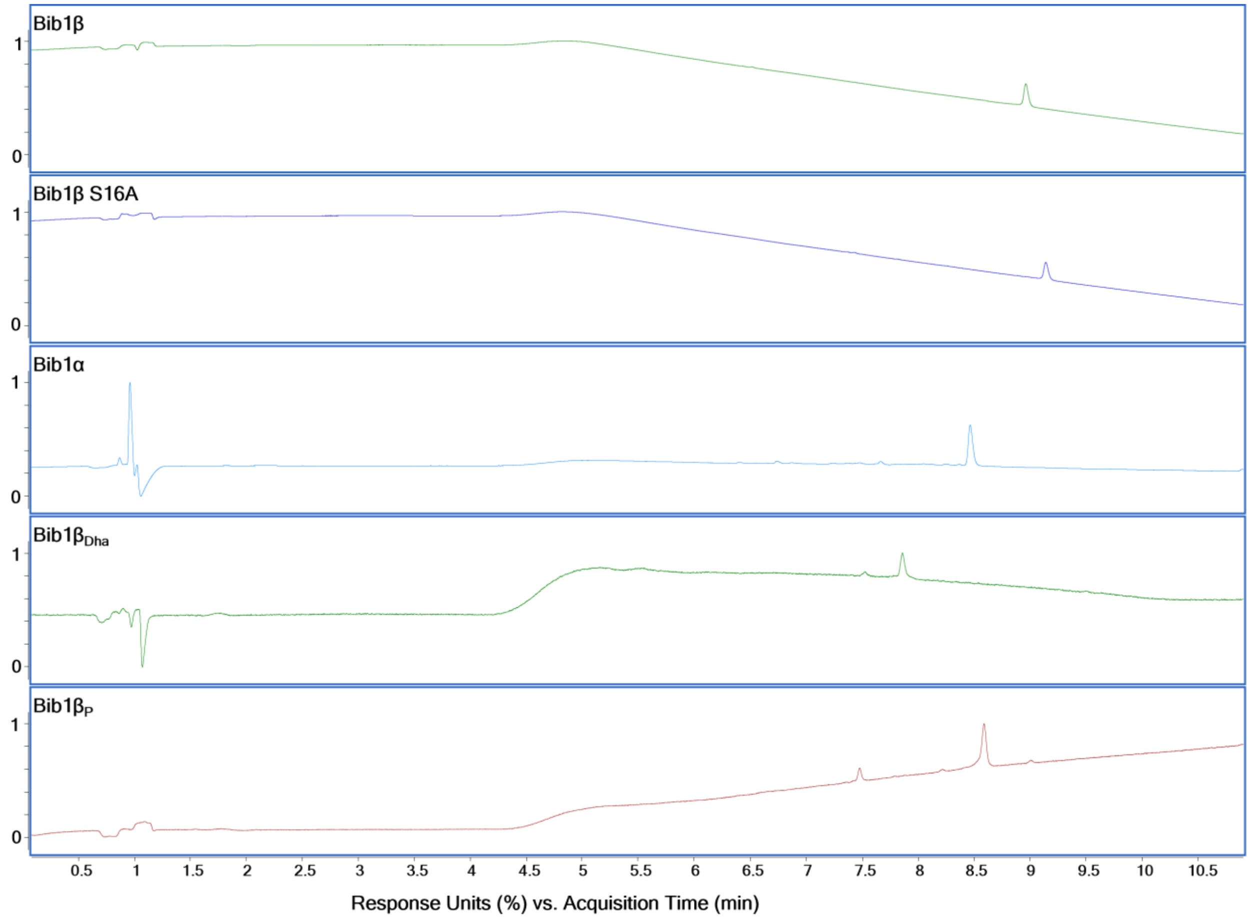

Fig. S7. Reversed phase-HPLC traces of purified Bib1α, Bib1β, Bib1β$_{P}$, Bib1β$_{Dha}$, and Bib1β S16A. Traces were collected using the conditions described in the High-Resolution Tandem Mass Spectrometry section of the Experimental.

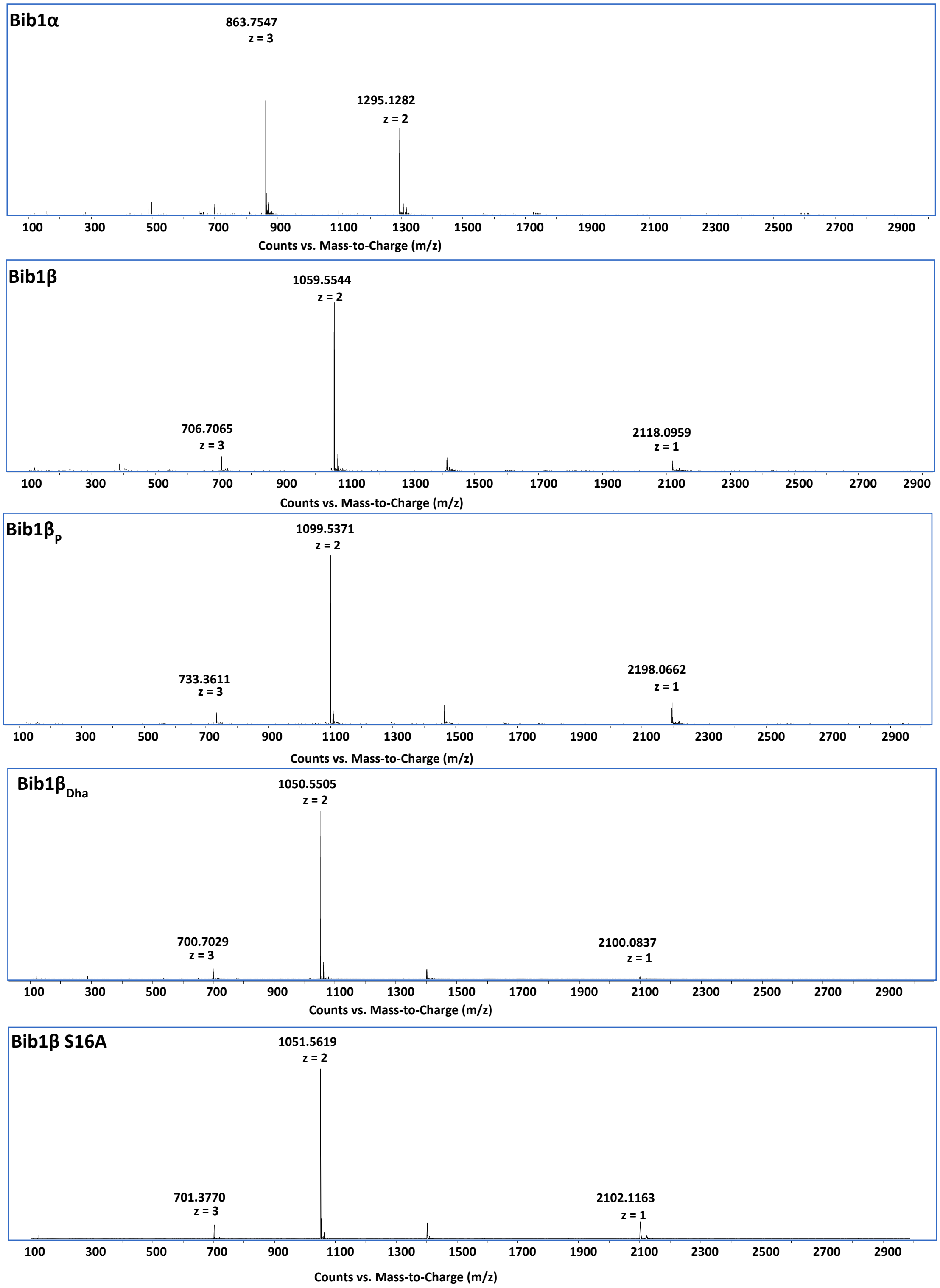

Fig. S8. HRMS spectra of purified Bib1α, Bib1β, Bib1β_P, Bib1β_{Dha}, and Bib1β S16A. HRMS data was collected using the conditions described in the High-Resolution Tandem Mass Spectrometry section of the Experimental. For calculated m/z values, see Table S5.

Table S5. Comparing high resolution m/z to expected m/z for Bib1α, Bib1β, Bib1β_P, Bib1β_{Dha}, and Bib1β S16A

Peptide	Expected m/z $[M+2H]^{2+}$	Observed m/z $[M+2H]^{2+}$	Δ (ppm)
Bib1α	1294.6233	1294.6255	2.2
Bib1β	1059.0497	1059.0559	6.2
Bib1β_P	1099.0329	1099.0344	1.5
Bib1β_{Dha}	1050.0445	1050.0485	4.0
Bib1β S16A	1051.0523	1051.0603	8.0

References

1. O. P. Kuipers, P. G. G. A. de Ruyter, M. Kleerebezem and W. M. de Vos, *J. Biotech.*, 1998, **64**, 15-21.
2. T. Mascher, S. L. Zimmer, T. A. Smith and J. D. Helmann, *Antimicrob. Agents Chemother.*, 2004, **48**, 2888-2896.
3. K. Held, E. Ramage, M. Jacobs, L. Gallagher and C. Manoil, *J. Bacteriol.*, 2012, **194**, 6387–6389.
